# Novel, Simple and Low-Cost Preparation of Ba-Modified TiO_2_ Nanotubes for Diclofenac Degradation under UV/Vis Radiation

**DOI:** 10.3390/nano11102714

**Published:** 2021-10-14

**Authors:** Mario Bohač, Tihana Čižmar, Vedran Kojić, Jan Marčec, Krunoslav Juraić, Ivana Grčić, Andreja Gajović

**Affiliations:** 1 Ruđer Bošković Institute, Bijenička cesta 54, 10000 Zagreb, Croatia; mario.bohac@irb.hr (M.B.); tcizmar@irb.hr (T.Č.); vkojic@irb.hr (V.K.); kjuraic@irb.hr (K.J.); 2 Faculty of Geotechnical Engineering, University of Zagreb, Hallerova aleja 7, 42000 Varaždin, Croatia; jan.marcec@gfv.hr (J.M.); igrcic@gfv.hr (I.G.)

**Keywords:** Ba-modification, transparent TiO_2_ nanotubes, thin film, photocatalysis, diclofenac

## Abstract

A novel low-cost synthesis of barium-modified TiO_2_ nanotube (TNT) arrays was used to obtain an immobilized photocatalyst for degradation of diclofenac. TNT arrays were prepared by electrochemical anodization of titanium thin films deposited on fluorine-doped tin oxide (FTO) coated glass by magnetron sputtering, ensuring transparency and immobilization of the nanotubes. The Ba-modifications were obtained by annealing solutions of Ba(OH)_2_ spin coated on top of TNT. Three different concentrations of Ba(OH)_2_ were used (12.5 mM, 25 mM and 50 mM). The crystalline structure, morphology and presence of Ba were characterized by X-ray diffraction, scanning electron microscopy and energy dispersive X-ray spectroscopy, respectively. Ba-modified TiO_2_ nanotubes (BTNT) were tested for photocatalytic degradation of diclofenac under UV/Vis radiation and it was proven that all of the Ba-modified samples showed an increase in photocatalytic activity with respect to the unmodified TNTs. The most efficient photocatalyst was the sample prepared with 25 mM Ba(OH)_2_ which showed 90% diclofenac degradation after 60 min. This result was in agreement with cyclic voltammetry measurements that showed the largest increase in photo-oxidation current densities for the same sample due to the increased generation of ^•^OH radicals obtained by a more efficient photogenerated charge separation.

## 1. Introduction

Titanium (IV) oxide is well known for its chemical and mechanical stability and non-toxicity. It is used in pigments, food additives, ceramics, cosmetics, self-cleaning surfaces, batteries, photocatalysis, photovoltaics, sensors, etc. [1]. As a metal oxide semiconductor, its uses in photocatalysis are extensively researched due to the fact that TiO_2_ is strongly oxidizable, chemically and biologically inert and affordable [2]. Recently, nanostructured TiO_2_ has almost completely taken the scientific focus off bulk TiO_2_ because of the large increase in its specific surface area. Nanostructuring TiO_2_ results in increased photo-induced reactions, light absorption, photogenerated charge carrier densities, photoreduction [3] and facilitates increased contact of the material and its surroundings [4] (e.g., with pollutants in photocatalysis). There are different nanostructured TiO_2_ formations varying from nanoparticles, nanotubes, nanorods, nanowires, nanobelts, nanoporous structures, etc. [5]. TiO_2_ nanotubes (TNT) are a one-dimensional (1D) nanostructure that can be prepared by various methods [6]. They are popular because of their low-cost fabrication, high surface to volume ratio, and their tubular elongated shape that facilitates charge transport along the axial direction [7].

One-dimensional TiO_2_ nanostructures in general have several critical issues concerning use in photocatalysis. Wide band gaps (*E*_g_) that vary among the crystal polymorphs (anatase 3.2 eV, rutile 3.0 eV) limit absorbance of the whole solar spectrum to a small part (3–5%) and fast rates of photogenerated electron–hole pair recombination lead to decreased efficiencies regarding uses in photocatalysis. Properties of nanostructured TiO_2_ materials can be further improved upon by doping or decorating, in other words, forming heterostructures with other materials [7].

Regarding vertically aligned TiO_2_ nanotubes, the majority of the research has been done on TNT prepared by anodizing Ti foil, as it is a robust system with a simple and cheap synthesis route. By varying the anodization parameters [8,9] such as reaction duration, voltage, electrolyte temperature, electrolyte content and electrolyte type, etc., different TNT morphologies can be obtained. The downside of this method is the limited application, since the nanotubes are attached to the Ti foil, opaque by default. Different research groups [10,11,12] reported methods of controlled detachment of TNT after anodization. These methods open up more implementation possibilities for TNT (prepared from Ti foil) but detaching and shattering TNT may potentially be hazardous to the environment and to the laboratory team working with them. By using physical deposition techniques, e.g., magnetron sputtering, Ti thin films can be prepared on different substrates and later converted into transparent TiO_2_ nanotubes by electrochemical anodization [13]. The thin film nanotube preparation method is analogous to Ti foil electrochemical anodization, so nanotubes prepared by this two-step method are perpendicularly aligned and bound to the substrate limiting nanoparticulate emission into the environment. Depending on the deposition [14] and anodization parameters [15] TNT are mechanically highly stable. Physical deposition techniques make it possible to choose the substrate type for the Ti film deposition. Choosing transparent conductive oxide (TCO)-coated glass as the substrate opens up possibilities of using the TNT in photovoltaic systems [16], while usage in photocatalysis shows potential, as TNT prepared on quartz (with an added TCO top-layer) reactor walls could be used in closed systems with outside illumination. The photocatalytic properties of TNTs can be improved by various methods of doping, decorating or preparing heterostructures. There has been a huge effort of the scientific community in preparing modifications of TNT structures. To name some, there have been different research groups that have presented modifications with different nanoparticulated noble metals like Au [17], Ag [18] and Pt [19] to improve photocatalytic properties of TNT. This practice of noble metal modification is standard and well known, but the method’s drawback is the cost of the starting materials and/or methods of synthesis. Cheaper alternatives are modifications with other metals like Cu [20,21], Fe [22] or metal oxides like CuO [23,24], ZnO [25,26], etc.

Barium, an alkaline earth metal, has potential to be a new cheap alternative material for TiO_2_ modification that is not that prevalent in literature with only a few mentions for photocatalytic use. It has been reported in the work of Shahmoradi et al. [27]. that Ba-doping increases the photocatalytic properties of TiO_2_ nanocrystals in the case of Acid Red 18 organic dye degradation, while, in the work of Hussain et al. [28], BaO increases the photocatalytic water splitting capabilities of TiO_2_ (P25) supported Pd nanoparticles. In these papers it was reported that doping influences the photoactivity of the photocatalyst by changing the absorption properties of the material and that BaO promotes charge separation and photocatalytic activity by raising the Fermi level. 

To the best of our knowledge, there has not been any research conducted on the photocatalytic degradation of active pharmaceutical ingredients (API) using Ba-modified TiO_2_ nanotubes, which could be an important future implementation because of the proposed low-cost fabrication.

As the model API pollutant, we chose diclofenac (DIC) for this work, which is a non-steroidal anti-inflammatory drug used to treat pain and inflammatory diseases. It is regularly detected in surface waters and in industrial wastewater due to its limited biodegradation [29], where the concentration of DIC varies between 0.44 and 7.1 µg/L [30]. Since the traditional water treatment processes are insufficient for the removal of this organic pollutant, emphasis should be put on technologies that will completely eliminate DIC from water streams. Advanced oxidation processes (AOPs) are a logical choice. The process includes the generation of powerful redox species, mainly the hydroxyl radicals (^•^OH), which are able to oxidize organic compounds [31]. For example, Boukhatem et al. [32] used mont-La (6%)-Cu_0.6_Cd_0.4_S nanocomposite photocatalysts for photo-degradation of DIC aqueous solutions under NUV-Vis irradiation and reported complete removal of DIC (92%) and a very high total organic carbon (TOC) removal (67%). Bi et al. [33] report on molecularly imprinted TiO_2_ on the surface of TiO_2_ particles with a diclofenac template. They showed the molecularly imprinted TiO_2_ had larger adsorption capacity, better selectivity and higher photodegradation performance for diclofenac than non-imprinted TiO_2_. Elangovan et al. [34] prepared TiO_2_-CdS heterojunction photocatalysts using hydrothermal synthesis and showed complete photodegradation of DIC within 4 h of irradiation. Mugunthan et al. [35] synthesized TiO_2_–SnO_2_ mixed-oxide catalysts and showed effective complete mineralization of DIC with a maximum TOC removal of 90% achieved under ultraviolet irradiation.

We showed efficient degradation using Ba-modified TNTs due to the low cost and simplicity of the syntheses. Therefore, in this study we prepared Ba-modified TiO_2_ nanotubes (BTNT) synthesized by a novel, simple and low-cost method. The samples were prepared by annealing spin coated water and ethanol mixed solutions (equi-voluminous) of Ba(OH)_2_ differing in concentrations on top of TiO_2_ nanotubes obtained by anodizing magnetron sputtered Ti thin films. It was observed that all of the Ba-modified TiO_2_ nanotubes (BTNT) samples showed better photocatalytic performances than unmodified TNT. The observed photocatalytic properties were correlated with structural and electrochemical properties of prepared samples. In order to investigate the crystalline structure and morphology of the BTNT photocatalysts, X-ray diffraction (XRD), field emission scanning electron microscopy (FE-SEM) and energy dispersive X-ray spectroscopy (EDS) were used. To investigate the electrochemical properties of the samples, and to provide insight into the photocatalytic mechanism, cyclic voltammetry (CV) was used.

## 2. Materials and Methods

### 2.1. Ba-Modified TNT Heterostructure Preparation

TiO_2_ nanotube (TNT) arrays were prepared by anodizing Ti thin films prepared by radio frequency (RF) magnetron sputtering on fluorine-doped tin oxide (FTO, Sigma-Aldrich Ltd., St. Louis, MO, France) coated glass substrates. Prior to magnetron sputtering, the FTO substrates were ultrasonically cleaned in acetone (99.8%, VWR Llc., Rosny-sous-Bois, France) and isopropanol (99.8%, VWR Llc., France) for 10 min, each, then rinsed with water and ethanol (EtOH, abs. 99.9%, Gram Mol Ltd., Zagreb, Croatia), dried with a stream of N_2_ and finally cleaned for 15 min in a UV-O_3_ cleaner (Ossila UV ozone cleaner E511). The sputtering conditions were kept constant for all the samples. Prior to Ti deposition, the base pressure of the magnetron vacuum chamber was evacuated down to 1.0 × 10^−6^ mbar. For the deposition process a steady flow of Ar (working gas) was pumped into the chamber at a rate of 20 sccm, raising the chamber pressure up to 5.0 × 10^−3^ mbar. A 2′′ diameter titanium target (Ti, 99.995%, Kurt J. Lesker Ltd., Jefferson Hills, PA, USA) was used and the deposition process lasted for 1 h at a set RF sputtering power of 150 W. To obtain the thin film TNT, the Ti samples were anodized in an ethylene glycol (99.7%, VWR Llc., France) electrolyte containing 0.6 wt% ammonium fluoride (NH_4_F, for analysis -ACS, Carlo Erba Reagents Srl., France) and 2.0 vol% water at 40 V for about 3 min. The anodization was stopped when the electric current values (mA) suddenly rose and the samples became semi-transparent. The sudden rise of current indicates that the electrolyte has reached the conducting FTO layer and that the anodization needs to be stopped to prevent further etching and delamination of the TNT. The anodization process was carried out at room temperature in a two-electrode reactor with a platinum cathode (99.9%, Sigma-Aldrich Ltd., St. Louis, MO, USA) and a Ti thin film anode (working electrode). The electrodes were set 1.5 cm apart and the reactor was filled with 18 mL of the electrolyte. After anodization the TNT thin films were rinsed with water and ethanol thoroughly and dried with N_2_ stream. To increase the transparency of the TNT thin films and to obtain the TiO_2_ anatase crystalline phase, the samples were annealed at 450 °C for 2 h in air (heating rate 120 °C/h).

The Ba-modified TNT samples were prepared by a low-cost and simple method of spin coating in which the anatase TNT thin films were used as a template for Ba-modification. Prior to Ba-modification the TNT films were heated for 60 min at 150 °C on a hot plate to remove any adsorbed water and gases and cleaned in an UV-O_3_ cleaner for 10 min to remove any remaining organic matter and to increase hydrophilicity [36].

The spin coating method included the deposition of an equi-voluminous ethanol and water (volume 1:1) solution containing 12.5, 25.0, 50.0 mM (Ba(OH)_2_∙8H_2_O, 97%, Alfa Aesar, Kandel, Germany), respectively. The method is similar to the synthesis of CaTiO_3_ in the work of Liu et al. [37]. The solutions were stirred for 1 h at 70 °C to promote dissolution of Ba(OH)_2_ and prior to spin-coating the solutions were filtered through a 0.25 µm PTFE (polytetrafluoroethylene/TEFLON) filter to remove any large undissolved crystals. No filtrate volume was discarded for filter saturation and immediately after filtration 60 µL of the warm solution was dropped onto the samples. After a waiting period of 10 s, the samples were spun at 3000 rpm for 20 s (static spin coating). The samples were then dried for 10 min on a hotplate set at 150 °C and later annealed in a tube furnace at 450 °C for 2 h (heating rate 120 °C/h). Annealing was conducted to promote the interaction between Ba(OH)_2_ and the TiO_2_ nanotubes to prevent potential modification leeching during photocatalysis measurements. The temperature of 450 °C was chosen as it would not disrupt the crystallinity of the anatase (TiO_2_) phase which is the preferred phase for photocatalysis.

The prepared samples are denoted as: TNT for unmodified TiO_2_ nanotubes, and 12.5 mM_BTNT, 25 mM_BTNT and 50 mM_BTNT, respectively, for the three different Ba-modified TNT samples.

### 2.2. Structural and Morfological Characterization 

Morphologies of the samples were investigated by field emission scanning electron microscopy (FE-SEM), model JSM-7000F manufactured by JEOL Ltd., Tokyo. Japan using an accelerating voltage of 10 kV, equipped with an energy dispersive X-ray spectrometer (EDS), EDS/INCA 350 (energy-dispersive X-ray analyser) manufactured by Oxford Instruments Ltd., which was used to prove the presence of barium in the samples.

The presence of Ba-species and anatase TiO_2_ was investigated by the means of X-ray diffraction analysis (XRD) using the Siemens D5000 diffractometer equipped with a Cu anode, Goebel mirror and graphite monochromator in front of the point detector. The experiments were performed in grazing incidence geometry with an incidence angle of 1.3° with respect to the film surface plane. 

### 2.3. Cyclic Voltammetry Measurements

The cyclic voltammetry (CV) measurements were performed in a three-electrode system. TNT or the BTNT were used as the working electrodes, silver–silver chloride electrode was chosen as the reference electrode and platinum wire as the counter electrode. The measurements were performed on a Keithley Model 2450-EC instrument. The electrolyte used for all experiments was a water solution containing 100 mmol/L potassium sulphate and 10 mmol/L sodium hydroxide which was continuously degassed with N_2_. For all of the CV measurements, a potential scan rate of 50 mV/s was used. For the light photocurrent measurements, a Xe lamp was used and calibrated at the 1 sun condition, utilizing 100 mW/cm^2^ of irradiance. To test if the oxygen had been removed (which is visible as no reduction peaks of O_2_) and to ensure/prove stability of the photocatalysts, the CV measurements were conducted 5 times.

### 2.4. Photocatalytic Activity 

#### 2.4.1. Photocatalytic Measurements

Photocatalytic activity tests were performed in a continuous flow reactor, i.e., small photocatalytic cell (SPC). The experimental procedure was previously described in detail [38]. Unmodified and Ba-modified TNT photocatalysts were placed at the bottom of the photocatalytic cell (*V* = 30 mL) perpendicular to the source of irradiation. A full-spectrum compact fluorescent bulb with high UVB intensity (JBL Reptile Desert UV, 15 W, 6500 K) was used as the irradiation source. Isoactinic conditions were obtained by placing the bulb in a special conical housing with a reflective inner surface. The intensities were measured at the samples’ surface by UVP UVX radiometer (*I*_UVB_ = 1.35 mW/cm^2^ and *I*_UVA_ = 2.45 mW/cm^2^) and by an HT204 solar power meter (*I*_Vis_ = 2.52 mW/cm^2^). The working solution of diclofenac (DIC) (99.9%, Sigma-Aldrich, St. Louis, MO, USA) was prepared by mixing together 15 mg of DIC and 1 L of deionized water (*C*_0_ = 15 mg/dm^3^). Prior to photocatalytic experiments, working solutions were recirculated (*Q* = 120 mL/min) over the samples in the dark for 15 min in order to achieve the adsorption–desorption equilibrium. Measurements were performed in time interval from 0 to 60 min. For each irradiation time (0 min, 20 min, 40 min and 60 min), three parallel samples of the aqueous solution (*V* = 50 µL) were taken from the SPC. To study the photolysis of diclofenac, control experiments without photocatalyst were performed. All experiments were performed twice with the same samples to examine the stability of the photocatalysts and to discard possible experimental errors.

#### 2.4.2. HPLC-UV Analysis

Diclofenac for standard preparation was purchased from Sigma-Aldrich (99.9%). MiliQ® water (18.2 MΩ/cm; purified by MiliQ water purification system (Millipore, Bedford, MA, USA)) with analytical-grade formic acid (FA) (Acros Organics, Geel, Belgium) and HPLC gradient-grade methanol (J.T. Baker, Center Valley, USA) were used for mobile phase preparation. Stock solution of 1H-benzotriazole was prepared as 1 mg/mL solution in MeOH. Working solutions were prepared from stock solution as 10 μg/mL and 1 μg/mL solution, respectively, by dilution with MiliQ water. The calibrants were prepared by further dilution of working solutions with MiliQ water in the range of 0.5–50 μg/mL. 

HPLC-UV analysis was carried out using an Agilent Technologies 1200 series HPLC system equipped with a binary pump, a vacuum membrane degasser, an automated auto sampler and a DAD detector (Agilent Technologies Inc., Palo Alto, CA, USA). The separation was performed on Synergi Fusion-RP 80 Å column (150 mm × 2 mm, 4 μm particle size) (Phenomenex, Torrance, CA, USA). Solvents for the analysis were 0.1% formic acid (FA) in water (solvent A) and methanol (solvent B). The gradient was applied as follows: 0 min 75% A, 0–3 min 75% A–50% A, 3–10 min 50% A–10% A, 10–15 min 10% A, 15.1–20 min 75% A. Flow rate was 0.3 mL/min. Sample injection was 5 μL. Retention time of diclofenac was 12.6 min. The ultraviolet detector was adjusted at 258 nm for absorption measurement. The calibration curve was obtained by linear regression; the peak area obtained at 258 nm was plotted versus the analyte concentration. Least-squares linear regression gave Spearman correlation coefficients of *R*^2^ = 0.9992 with the regression lines of *y* = 21.017*x* − 7.8272. During analysis, all instrumental blank samples were negative and areas of calibration samples at 5 μg/mL were repeatable. All data acquisition and processing was performed using Agilent MassHunter software.

## 3. Results and Discussion

### 3.1. Field Emission Scanning Electron Microscopy and Energy Dispersive X-ray Spectroscopy

**Figure 1:**
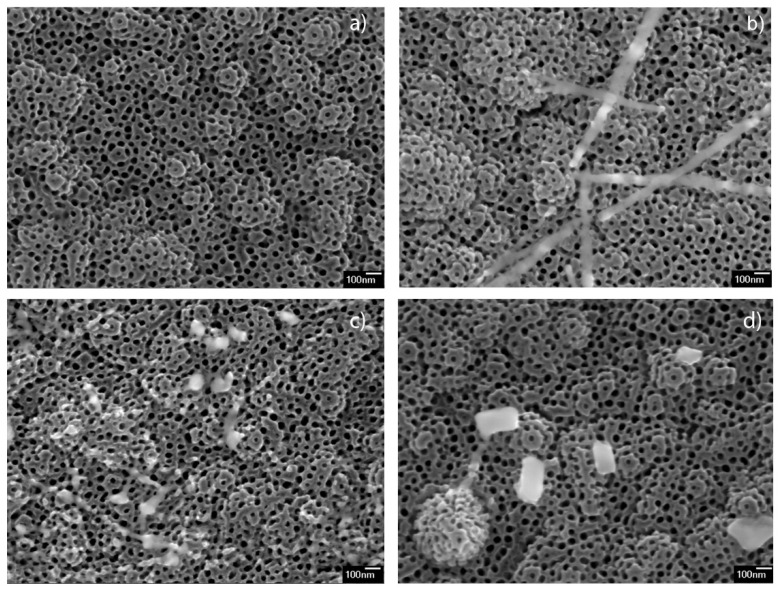
FE-SEM 50 kx magnification micrographs of (**a**) unmodified TNT, and Ba-modified samples: (**b**) 12.5 mM_BTNT, (**c**) 25 mM_BTNT, (**d**) 50 mM_BTNT.

The morphologies of the Ba-modified samples are shown in Figure 1. The images display the presence of TiO_2_ nanotubes array in all the samples, having the same morphology as non-modified sample (Figure 1a). The sample modified with 12.5 mM (12.5 mM_BTNT) additionally contained small amounts of nanoparticles, smaller than 10 nm, and only a few nano-strips at the surface of the nanotube arrays (Figure 1b). As can be seen in Figure 1c,d, the samples prepared using higher Ba(OH)_2_ precursor concentrations (25 mM_BTNT and 50 mM_BTNT) showed higher amounts of nano-objects that are sporadically dispersed in form of nano-cubes, irregularly shaped nano-strips and very small nanoparticles at the TiO_2_ nanotube array surface. 

**Figure 2:**
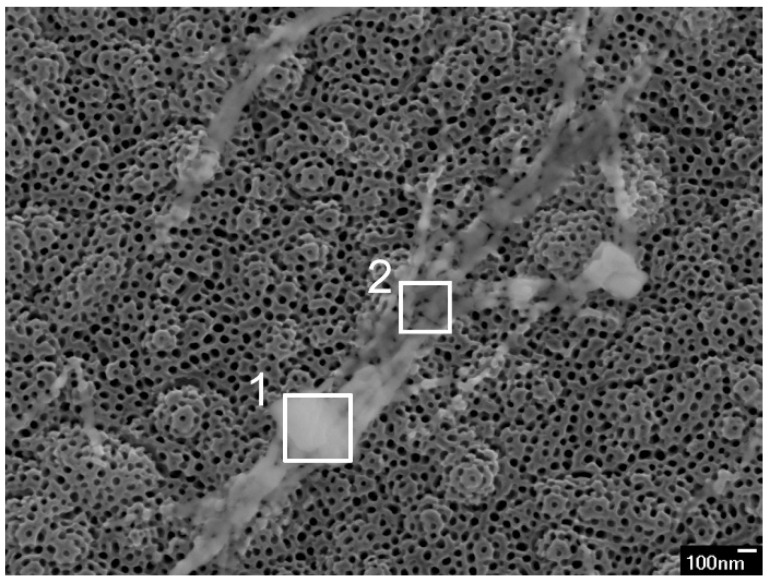
Areas of the nano-objects, obtained in a 50 mM_BTNT sample, measured by EDS.

EDS measurements for the unmodified sample showed no presence of barium, while the average loading of barium on each modified sample is presented in Table 1. In the case of the modified samples, EDS measurement of individual (nano-)objects indicated that the formed nano-strips and nano-cubes contain larger atomic percentages of barium compared to the TNT environment (Table 2). In the 50 mM_BTNT sample, even larger objects were observed, as is shown in Figure 2. EDS of the cube (200 × 200 nm) presented in Figure 2 (area 1) and showed even 8.7 at.% of Ba (73.5 at.% O and 17.8 at.% Ti) since the EDS was obtained at larger magnification. EDS of the strips in Figure 2 (area 2) also showed a larger amount of Ba (2.6 at.% Ba, 68.9 at.% O and 28.5 at.% Ti) since these strips were almost a micrometre in size. On the other hand, EDS measurements obtained on nano-sized cubes showed 1.6 at.% of Ba on average, while measurements on nanostrips show 1.0 at.% of Ba (Table 2). EDS measurements also indicate a very small amount of Ba dispersed at the surface of TNTs (Table 2).

It should be mentioned that smaller at.% of barium was observed in EDS spectra of nano-objects (in relation to titanium and oxygen) in comparison with spectra of larger objects. That could be explained keeping in mind the main feature of EDS measurement, i.e., it is expected to obtain a higher amount of Ti and O in the spectra due to ~1 μm penetration depth, so titanium and oxygen from nanotubes are prevalent in the spectra. 

It could be concluded, both from SEM observations and EDS measurements that, after spin coating and heating, Ba(OH)_2_ mainly formed Ba-rich nano-objects, sporadically distributed on the TNT surface. The surface of TNT in all Ba-modified samples was covered with only a very small amount of barium, almost under the detectable limit of EDS measurements. Larger sized objects (>100 nm), Ba-rich cubes and stripes, were observed only in the 50 mM_BTNT sample by systematic inspection of all the surfaces by SEM.

### 3.2. X-ray Diffraction Measurements

**Figure 3:**
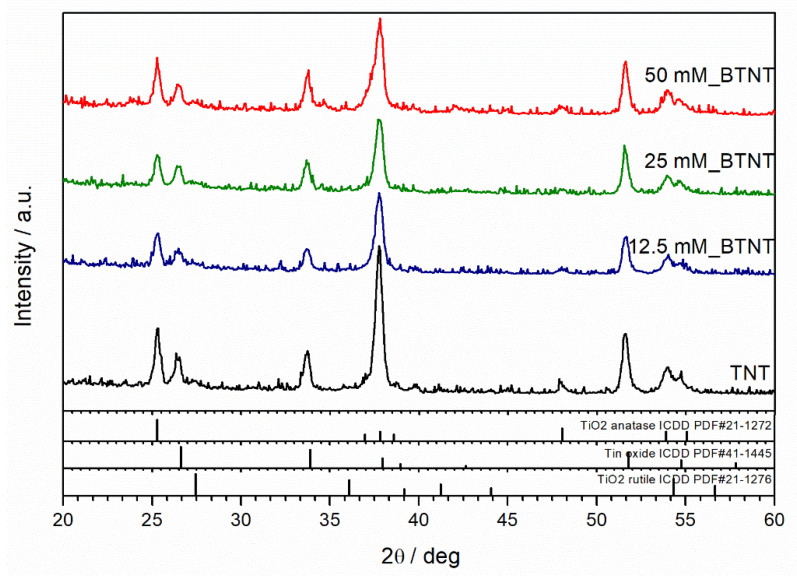
XRD diffractograms for Ba-modified TNT samples (12.5, 25.0, 50.0 mM).

XRD was used to resolve the phase composition and crystalline structure of as-prepared samples. X-ray diffraction patterns of unmodified and Ba-modified nanostructured TiO_2_ samples (12.5 mM_BTNT, 25 mM_BTNT and 50 mM_BTNT) are shown in Figure 3. The XRD analysis demonstrated that anatase (ICDD PDF#21–1272) is the main crystalline phase in all samples. Fluorine-doped tin oxide (FTO) (ICDD PDF#41−1445) diffraction peaks are also visible, originating from the FTO film on the glass substrate, as well as the small amount of rutile phase (ICDD PDF#21−1276). However, the addition of Ba-compounds did not affect the crystalline structure of TiO_2_ indicating that Ba-compounds are amorphous or highly dispersed on the TiO_2_ surface.

### 3.3. Cyclic Voltametry Measurements

**Figure 4:**
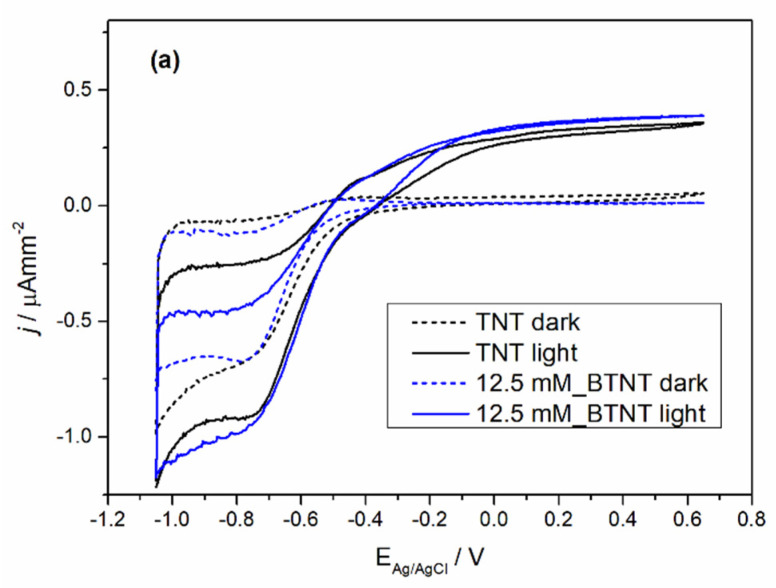

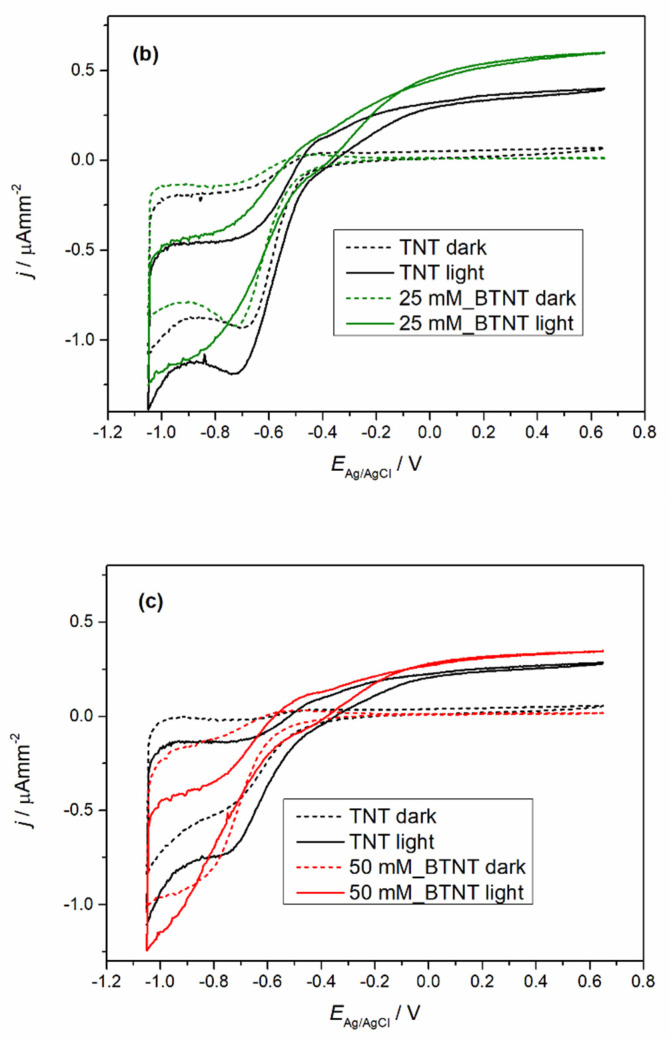
Voltammograms of unmodified TNT and Ba-modified TNT samples: (**a**) 12.5 mM_BTNT, (**b**) 25 mM_BTNT and (**c**) 50 mM_BTNT in dark and light measurements.

The prepared samples were characterized by cyclic voltammetry (CV) in the dark and under 1 sun conditions (light), before and after Ba-modification to exclude the current density variation that could occur between different TNT films. The obtained voltammograms are shown in Figure 4.

In all the cases of Ba-modified TNT samples, it can be observed that after modification, light measurements show an increase in photo-oxidation current densities and a decrease in oxidation currents in the dark measurements. The dark measurement current density drop could be attributed to surface coverage as the result of the Ba-modification. The surface coverage changed the chemical nature of the solid–liquid interface which leads to a higher energy barrier for the oxidation process in the dark. Additional evidence supporting this claim of the surface coverage can be seen in the trend of Ti^4+^ reduction to Ti^3+^ peak loss in the reduction currents (in our case the peak around −0.7 V vs. Ag/AgCl reference electrode) [39]. With the increase in the Ba(OH)_2_ precursor concentration, the reduction peak became less pronounced. Under light measurements, all of the samples show an increase in oxidation current values. In order to evaluate the extent of the increase in the photo-oxidation current densities, every sample was measured before modification (TNT) and after modification (BTNT). The increase in the current density at the voltage values of 0.65 V was compared by using the following equation (Equation (1)):

(1) $$Current\;increase=\frac{j_{light}-j_{dark}}{j_{dark}}$$

As is shown in Table 3, Ba-modifications lead to a significantly larger current increase in comparison to unmodified TNT films, with the largest increase for the sample 25 mM_BTNT. These results show strong evidence that the Ba modification increased the number of photogenerated holes in the valence band, which in term resulted in the increase in the oxidation currents and was directly impacted by the amount of covered TiO_2_ surface. At the concentration of 25 mM, there was an optimal amount of Ba on the surface without interference or blocking of the TiO_2_–liquid interface. For lower concentrations this effect was less pronounced (sample (a) 12 mM_BTNT). However, by increasing the concentration (sample (c) 50 mM_BTNT), the surface blockage became more pronounced since less TiO_2_ was exposed on the interface, leading to fewer TiO_2_ photogenerated valence holes which can oxidize water molecules. 

These results were important for interpreting photocatalysis results as the increase in photo-oxidation current densities implies the increase in generated ^•^OH radicals that drive photocatalysis.

As stated in the experimental part, CV measurements were conducted five times. Between the repeated CV measurements, no changes in the voltammograms were noticed, thus proving that there were no changes or degradation of the samples.

### 3.4. Photocataltic Activity

**Figure 5:**
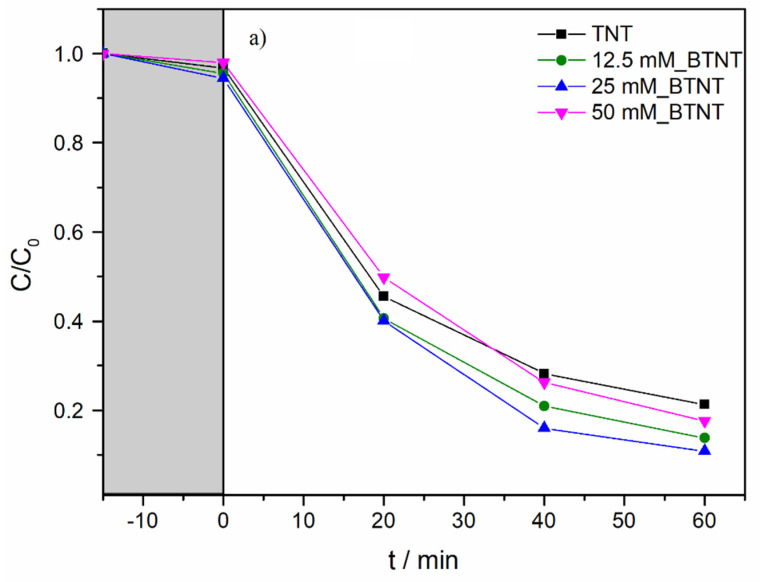

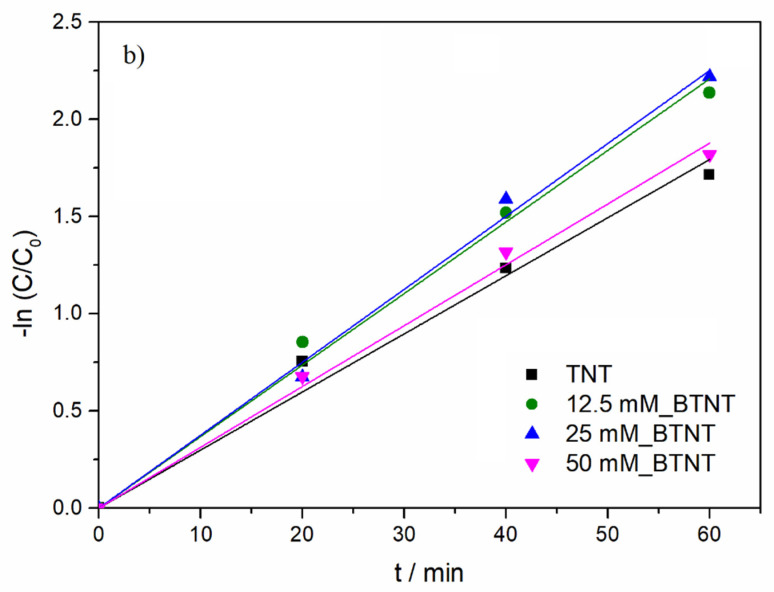
(**a**) Adsorption in the dark (interval from −15 to 0 min; grey area) and photocatalytic degradation of DIC under UV/Vis for Ba-modified TNT photocatalysts with different Ba(OH)_2_ concentrations. (**b**) Photocatalytic activities of TNT and Ba-modified TNT for photodegradation of DIC aqueous solution under ambient conditions.

In order to investigate the photocatalytic activity of the prepared photocatalysts, and to elucidate the role of the Ba-modifications of TNT, SPC and a full-spectrum compact fluorescent bulb with high UVB intensity were used. Diclofenac (DIC) was used as a model pollutant. The experimental observations concerning the decrease in DIC concentration during photocatalytic experiments are shown in Figure 5. Before all the photocatalytic experiments, it was necessary to achieve adsorption–desorption equilibrium; therefore, all the solutions containing DIC and immobilized catalyst samples were stirred in the dark for 15 min (Figure 5, grey region). 

Modelling of the experimental data (DIC concentration as a function of UV/Vis illumination time) was performed using a pseudo-first order kinetics model (Equation (2)):

ln(*C*/*C*_0_) = −*kt* (2)

where *C*_0_ is an initial DIC concentration (*C*_0_ = 15 mg/L), *C* represents DIC concentration at time *t*, while *k* is the reaction rate constant. 

As shown in Figure 5b and Table 4, under UV/Vis radiation, the DIC degradation rate exhibited the following order: 25 mM_BTNT > 12.5 mM_BTNT > 50 mM_BTNT > TNT. Moreover, two Ba-modified photocatalysts (12.5 mM_BTNT and 25 mM_BTNT) exhibited higher photocatalytic efficiency in comparison to the unmodified reference sample (TNT), while the 50 mM_BTNT had similar photocatalytic activity as the unmodified TNT sample. It should be mentioned here that, in our previous study [40], using numerical comparative quantification and the kinetic model, we demonstrated the similar photocatalytic activity of TiO_2_ nanotube arrays and commercial TiO_2_ P25 for degradation of API in each spectral range (UV and visible).

In comparison to TNT, the degradation efficiencies of the 12.5 mM_BTNT and 25 mM_BTNT photocatalysts were 23% and 28% higher than the reference TNT sample, respectively. This demonstrates that it is crucial to find an optimal concentration of Ba(OH)_2_ precursor (in our case 25 mM). The 25 mM_BTNT sample was the most efficient in DIC removal since 90% of diclofenac was degraded after only 60 min, while other authors report much longer removal times and rates using different TiO_2_-based photocatalysts, e.g., in the case of imprinted DIC selective TiO_2_ photocatalyst [41] and Cu nanoparticle modified TNTs [42]. 

The obtained photocatalytic results were also in agreement with cyclic voltammetry results concerning the highest increase in photo-oxidation current densities in the 25 mM_BTNT sample. For the sample modified with the highest Ba(OH)*_2_* concentration (50 mM), the reduced photocatalytic activity could be due to two effects: (1) the shielding effect, which reduced the TiO_2_’s ability to absorb light irradiation, decreasing the generation of electrons and holes, and (2) the high concentration of Ba-species trapping more electrons, which were then unavailable to reduce oxygen and produce hydroxyl radicals responsible for the degradation of diclofenac. 

Based on the experimental photocatalysis results and CV measurements, it could be concluded that Ba-modification resulted in an increase in photocatalytic activity, which is attributed to more efficient photogenerated charge separation (Figure 5b). The rise in photo-oxidative current densities (CV measurements) for Ba-modified samples, with respect to the unmodified samples, implies higher concentrations of generated ^•^OH radicals which confirms our hypothesis of more efficient charge separation. Higher concentrations of ^•^OH radicals present in the photocatalytic reactors result in faster DIC degradation as they are the main pathway of oxidizing/degrading organic pollutants. Our findings are in agreement with Elangovan et al. [34] who studied effect of different scavengers (potassium iodide (KI), methanol and sodium azide (NaN_3_)), and concluded that photocatalytic efficiency was significantly reduced with the addition of 0.01 M KI or 10% methanol (scavengers for both h^+^ and ^•^OH radicals) to 100 mL of DIC solution (*C*_0_ = 20 mg/L), indicating that hydroxyl radicals are the primary active species in photocatalytic degradation of DIC.

Furthermore, we could speculate that under the experimental conditions, the surface of the TiO_2_ nanotubes (TNT) is positively charged which allows one of the oxygen atoms of the carbonyl group of DIC to chemisorb on the TNT surface. Based on the experimental results, the possible mechanism responsible for photocatalytic activity improvement of Ba-modified TiO_2_ nanotubes could be described as it follows: under UV/Vis radiation, electrons (e^−^) from TiO_2_ valence band (VB) are excited to the conduction band (CB), leaving hole (h^+^) in the VB, thus generating electron-hole (e^−^-h^+^) pairs. Part of photogenerated electrons in the CB can be easily transferred to the barium sites on the TiO_2_ surface; meanwhile, the photogenerated holes remain in the VB and, therefore, Ba-modifications improve charge carrier separation and, consequently, enhance the photocatalytic activity. Hydroxyl radicals and photogenerated holes are responsible for oxidation reactions, i.e., degradation of organic pollutants.

Tentative degradation pathways are proposed for the photocatalytic degradation of diclofenac based on the formation of hydroxy-derivatives before the complete mineralization of the starting molecule. Pérez-Estrada et al. [43] established the degradation pathway and identified 18 intermediates in two different degradation routes. The main one was based on the initial hydroxylation of the phenylacetic acid and subsequent formation of a quinone imine derivative which was the starting point for further multistep degradation involving hydroxylation, decarboxylation, and oxidation reactions. Calza et al. [44] studied diclofenac transformations through a mass spectrometry analysis and characterized several hydroxy- and dihydroxy-diclofenac derivatives which were transformed into chloro or hydroxyl-phenol derivatives. The degradation pathway and the kinetics were discussed in detail, which is in agreement with the work reported by Boukhatem et al. [32].

## 4. Conclusions

A novel low-cost and simple synthesis route was used for preparing Ba-modified transparent TiO_2_ nanotube arrays (TNT) which involved annealing spin-coated Ba(OH)_2_ (different concentrations) water and ethanol (1:1) solutions on top of the TNTs. The formation of Ba-species (nano-cuboid and nano-strip-like structures with irregular edges) on the surface of TNT was confirmed by SEM and EDS.

Two Ba-modified samples (12.5 mM_BTNT and 25 mM_BTNT) demonstrated improved photocatalytic degradation of diclofenac in respect to unmodified TNT. The sample modified with 25 mM Ba(OH)_2_ (25 mM_BTNT) improved the degradation rate of diclofenac ~30% in comparison to the unmodified reference sample, and also successfully degraded ~90% of DIC in the first 60 min of the photocatalytic experiment. These results were in agreement with cyclic voltammetry measurements that showed the largest increase in oxidation current was, indeed, for the same sample. Therefore, the enhanced photocatalytic activity is derived from a more successful charge carrier separation that increased the generated ^•^OH radical concentration, which is most responsible for photocatalysis.

## Figures and Tables

**Table 1:** The average actual percentages of Ba on each modified sample.

	Atomic %		
	12.5 mM	25 mM	50 mM
O	68.7 ± 0.4	69.1 ± 0.3	68.8 ± 0.8
Ti	30.7 ± 0.2	29.6 ± 0.3	29.1 ± 0.8
Ba	0.6 ± 0.3	1.3 ± 0.2	2.1 ± 0.8

**Table 2:** Atomic percentages of the present O, Ti, Ba elements in nanocube and nanostrip structures and in the background.

	Atomic %
Nanocubes	
O	69.2 ± 0.4
Ti	29.6 ± 0.4
Ba	1.6 ± 0.2
Nanostrips	
O	67.5 ± 0.5
Ti	31.5 ± 0.7
Ba	1.0 ± 0.3
Background	
O	68.0 ± 0.6
Ti	31.2 ± 0.6
Ba	0.8 ± 0.2

**Table 3:** Photo-oxidation current density increase for unmodified and modified TNT substrates.

Scheme	TNT Current Increase	BTNT Current Increase
(a) 12.5 mM_BTNT	5.64	32.99
(b) 25 mM_BTNT	4.61	47.98
(c) 50 mM_BTNT	4.03	20.45

**Table 4:** Photocatalysts overview with obtained corresponding rate constants *k* (pseudo-first order kinetic model).

	*k*/min^−1^	Rel. *k*	*R* ^2^
TNT	0.0298	1	0.9915
12.5 mM_BTNT	0.0368	1.23	0.9962
25 mM_BTNT	0.0381	1.28	0.9974
50 mM_BTNT	0.0312	1.05	0.9975

## Data Availability

Not applicable.
